# Hyperbaric oxygen therapy for post concussion symptoms: issues may affect the results

**DOI:** 10.1186/s13618-015-0033-3

**Published:** 2015-08-25

**Authors:** Qin Hu, Anatol Manaenko, Zhenni Guo, Lei Huang, Jiping Tang, John H. Zhang

**Affiliations:** Departments of Physiology and Pharmacology, Loma Linda University School of Medicine, 11041 Campus Street, Risley Hall, Room 219, Loma Linda, CA USA; Department of Neurosurgery, Loma Linda University School of Medicine, 11041 Campus Street, Risley Hall, Room 219, Loma Linda, CA USA

**Keywords:** Hyperbaric oxygen therapy, Post concussion syndrome, Clinical studies

## Abstract

Post concussion syndrome (PCS) is a set of symptoms succeeding in 25 % of mild traumatic brain injury (mTBI) patients. Hyperbaric oxygen therapy (HBOT) has been demonstrated as an effective method for treating acute and severe TBI, but its efficacy in PCS remains controversial. In this editorial, we reviewed the clinical studies of HBOT in PCS, summarized the limitations of these studies, and discussed the limitations: inappropriate Sham group using room air at 1.2 or 1.3 ATA; delayed HBO administration; subjective assessment methods; time point for outcome assessment and small sample size. We hope that our concerns will be helpful for future clinical studies of HBO therapy in TBI or other neurological disorders.

## Commentary

### Hyperbaric oxygen therapy for Post concussion Symptoms: issues may affect the results

Post concussion syndrome (PCS) is a set of symptoms succeeding in 25 % of mild traumatic brain injury (mTBI) patients [1]. The symptoms of PCS include headache, dizziness, neuropsychiatric symptoms, and cognitive impairments, and may continue for weeks, months, or a year or more. Hyperbaric oxygen therapy (HBOT) is an effective method for treating acute and severe TBI [2], however, effects of HBOT in the treatment of PCS has not been well established. By our current research in Pubmed, using key words “hyperbaric oxygen and post concussion syndrome”, we were able to identified seven articles to this topics. HBOT was effective in three [3–5] but failed in four studies [6–9]. The studies from Boussi-Gross R et al [3] and Harch PG et al [4, 5] showed HBOT (1.5 atmospheres absolute [ATA], 60 min once or twice daily for 40 sessions) significantly improved symptoms, cognitive abilities, and quality of life, with concomitant improvements in single photon emission computed tomography (SPECT) imaging. In the three positive studies, there were no control groups and they compared the data pre- and post-HBOT to get the conclusion. However, the results from the studies of David X. Cifu et al [6, 7], R. Scott Miller et al [8] and George Wolf et al [9] failed to prove the therapeutic effects of hyperbaric oxygen (HBO) in PCS after mild TBI. In these four prospective, randomized studies, the subjects were U.S. military service members, and received 30 to 40 sessions of either a sham or HBO (1.5–2.4 ATA, 60 min once daily) in the treatment of PCS after mild TBI; and the outcome was primarily assessed by the Rivermead Post-Concussion Symptoms Questionnaire (RPQ). In Dr. Miller’s study, the authors concluded that HBO is ineffective for the treatment of PCS, but the ritual of the intervention does. It has been shown through these various studies that the effects of HBOT in PCS are controversial. We believe that there are numerous inherent limitations in these studies, which contributed significantly to misinterpretation of the HBO effects. We want to discuss these limitations, and hope that our concerns will be helpful for future clinical studies of HBOT in TBI or other neurological disorders.

The usage of a correct placebo is a dilemma, which has been controversially debated in the field of HBO therapy. In Dr. Miller’s and Dr. Wolf’s studies, the sham group received a treatment with a pressurized room air at 1.2 ATA or 1.3 ATA [8, 9]. The authors have mentioned that the remarkable strengths of their studies are well-designed control groups. This allowed the participants to sense pressure increases with minimal biological function. Authors believed the improvement in HBO and Sham groups is due to the placebo effects from the intensive rituals of the repetitive chamber procedures, rather than oxygen effects. Of course, the placebo effects cannot be ruled out entirely; however, there are other arguments speaking against such interpretation of the observed results. Can room air at 1.2 ATA really serve as a proper sham-control due to it being “logically implausible to have a beneficial effect”? The “sham” control group was exposed to 1.2 ATA of room air, which is a 20 % increase in pressure and, that, theoretically, should increase plasma oxygen for about 30 % compared to normal pressure. Some case reports clearly demonstrate biological effects of even insignificant increases of air pressure in different tissues, including effects on the brain [10]. Similarly, randomized, controlled clinical trials have shown that room air at 1.3/1.2 ATA leads to meaningful improvements of neurological functions [9, 10]. Previous studies demonstrated that treatment with medical air under mild pressure could have a beneficial effect. Interesting enough, Miller et al. are aware of this finding and they have mentioned it in the “discussion” section. Both Miller and Wolf failed, however, to provide the result of blood plasma oxygen measurement of the sham group, one of the most imperative physiological parameter of this study, and failed to show the dose response of HBO in the patients. In the other three positive studies from Boussi-Gross R et al [3] and Harch PG et al [4, 5], they used self controlled case-series design method to overcome the placebo issue by comparing the results pre- and post-HBOT.

The optimal time for HBO administration is one of the crucial facts that determine its efficacy in TBI and other neurological diseases. The neuroprotective effects of HBO have all been achieved when intervention was administered during the acute phase, within hours after TBI [11–16]. The prolonged therapeutic time window of HBO was further investigated in animal models of TBI. Yang et al. showed HBO treatment decreased apoptosis and improved cognitive ability when it was given 6 hours after TBI in rats, while failed to work when given 60 days after TBI [16]. Wang and colleagues have demonstrated that multiple sessions of HBO (3 ATA hourly for 3 or 5 days) extended the time window of HBO compared to a single session up to 48 hours post-TBI and significantly reduced overall neurological deficit scores and neuronal apoptosis [17]. The data of these pre-clinic and clinic studies indicated that HBO is beneficial when it is applied in the early stage. Most PCS patients were treated more than one year after the onset of the most recent TBI. It would be more logical to start HBO treatments within a therapeutic time window established in preclinical study. Whether earlier HBO therapy affords clear benefits remains to be determined.

Objective and precise assessment methods are another challenge in the evaluation of the efficacy of HBO therapy in PCS patients. In most studies, the outcome was evaluated by the Rivermead Post-Concussion Symptoms Questionnaire (RPQ), Neurobehavioral Symptom Inventory and Automated Neuropsychological Assessment Metrics. All assessments are well established, however, they are all subjective performance evaluations. It is well known that RPQ displays several flaws in its implementation and in its ability to accurately reflect test-taker experience [18]. Interpretation and accuracy of the RPQ can vary widely due to self-administration and the various confounding variables involved, because it is sensitive to subjective patient memory, social desirability, stress, and other covariates such as personality factors and willingness to reveal problems, as are the two other methods. The authors relied totally on the self-administration assessments which is a weakness of these failed studies. Additionally, in many studies, participants in HBO and sham groups were given different medications on board and undergoing nonpharmacological interventions for post-traumatic stress disorder (PSD) treatment. These professional psychiatric treatments for PSD may contribute to, or account for, the elevated RPQ and mask the efficacy of HBO. The correlation between medication status and scores on any of these measurements should be studied in the future studies. In the three positive studies, the resolution of symptoms and signs of TBI/PCS were reflected in global and focal improvements in brain blood flow analyzed by SPECT. Even though SPECT imaging or electrophysiological measurements may not sensitive enough to detect abnormal in some patients with PCS, it is an objective assessment methods that can provide authentic evidences for HBO or sham interventions and allow a greater refinement of HBO application in mTBI.

Forth, the time point for outcome assessment may be one of the factors that affect the effects of HBOT. In this article, all the subjects of these seven studies were mild TBI patients with prolonged PCS at late chronic stage (three month to three years after the first TBI), and post-exposure assessments of HBOT were performed at 3 days [5], 1 week [4], 1 month [8], 6 weeks [9], 2 month [3], and 3 month [6, 7] after the last chamber exposure. The different time points for outcome assessments may be another contributor to the varied results. In addition, restoration of neurological deficits is a slow process, and TBI patients at the chronic late stage may need more time to demonstrate the effects of HBOT. Evaluation of the outcome 6 or 12 month after HBOT is much more preferable.

At last, the small sample size limits the power of these studies. In the three positive studies, the sample number is 24 in crossover group and 32 in HBOT group [3], 16 [4] and 1 [5] in HBOT group respectively. In other four studies, in Cifu’s study, the eligible participants in Sham, 1.5 ATA HBO and 2.0 ATA HBO groups were 21, 21, and 19. In Dr. Miller’s study, the numbers of patients Intent-to-Treat in HBO and Sham groups are 23, 21 respectively; and the actual number in HBO and Sham groups finishing the whole project are 11 and 13 respectively. The inadequacy of sample size might bias the results and lead to an incorrect conclusion.

HBOT has showed great potential in neuroprotection and neurogenesis, and the inefficacy of HBOT in PCS in these four clinic studies demands a cautious interpretation. The incorrect sham group, delayed treatment time, inappropriate outcome measurements, time point for outcome assessment and limited number of patients could contribute to misinterpretation of results and prevent a positive recommendation of HBO in PCS. We hope these key factors can be considered in the future clinical studies of HBO in mTBI and other neurological diseases.

## Abbreviations

PCS: Post concussion syndrome
mTBI: mild traumatic brain injury
TBI: Traumatic brain injury
HBOT: Hyperbaric oxygen therapy
SPECT: Single photon emission computed tomography
HBO: Hyperbaric oxygen
ATA: Atmospheres absolute
RPQ: Rivermead Post-Concussion Symptoms Questionnaire
PSD: post-traumatic stress disorder
